# Supplementary material for: Burden and resources in caregivers of people with multiple sclerosis: A qualitative study
Source: PLoS One. 2023 Apr 17;18(4):e0265297. doi: 10.1371/journal.pone.0265297 (PMC10109507; doi:10.1371/journal.pone.0265297)
Supplement: S1 File — (PDF) [file pone.0265297.s001.pdf]

FRONTESPIZIO PROTOCOLLO GENERALE

AOO: ASL_BO

REGISTRO: Protocollo generale

NUMERO: 0020652

DATA: 26/02/2021

OGGETTO: Risposta a: Quesito su progetto qualitativo e posizione CE - FATICHE E RISORSE NEI CAREGIVER DI PERSONE CON SCLEROSI MULTIPLA. UNO STUDIO QUALITATIVO

SOTTOSCRITTO DIGITALMENTE DA:

Carlo Descovich

CLASSIFICAZIONI:

- [01-02-05]

DOCUMENTI:

File	Firmato digitalmente da	Hash
PG0020652_2021_Lettera_firmata.pdf:	Descovich Carlo	933B881EAC6ED8EDB62282865D67C990A7C1EF25FF390B81B71512F93B438802
PG0020652_2021_Allegato1.pdf:		C0543DD574FF5A22A4B9E9D44273C47CB39CD6E9991101DDDB23CF2D25CCA4FB

L'originale del presente documento, redatto in formato elettronico e firmato digitalmente e' conservato a cura dell'ente produttore secondo normativa vigente.
Ai sensi dell'art. 3bis c4-bis Dlgs 82/2005 e s.m.i., in assenza del domicilio digitale le amministrazioni possono predisporre le comunicazioni ai cittadini come documenti informatici sottoscritti con firma digitale o firma elettronica avanzata ed inviare ai cittadini stessi copia analogica di tali documenti sottoscritti con firma autografa sostituita a mezzo stampa predisposta secondo le disposizioni di cui all'articolo 3 del Dlgs 39/1993.

SERVIZIO SANITARIO REGIONALE
EMILIA-ROMAGNA
Azienda Unità Sanitaria Locale di Bologna

UO Governo Clinico e Sistema Qualita' (SC)

Istituto delle Scienze Neurologiche
Istituto di Ricovero e Cura a Carattere Scientifico

Benini Stefano
stefano.benini@ausl.bologna.it

Benini Stefano (UO Governo Clinico e Sistema Qualita' (SC))
Babel di Benini Stefano (UO Governo Clinico e Sistema Qualita' (SC))

OGGETTO: Risposta a: Quesito su progetto qualitativo e posizione CE - FATICHE E RISORSE NEI CAREGIVER DI PERSONE CON SCLEROSI MULTIPLA. UNO STUDIO QUALITATIVO

Gent.mo,

si trasmette di seguito il documento relativo all'oggetto.

Cordiali saluti,
Carlo Descovich

Firmato digitalmente da:

Carlo Descovich

Responsabile procedimento:
Corrado Iacono

Corrado Iacono
UO Governo Clinico e Sistema Qualita' (SC)
0516584897
corrado.iacono@ausl.bologna.it

Azienda USL di Bologna
Sede legale: via Castiglione, 29 - 40124 Bologna
Tel +39.051.6225111 fax +39.051.6584923
Codice fiscale e partita Iva 02406911202

Il Comitato Etico di Area Vasta Emilia Centro della Regione Emilia-Romagna (CE-AVEC) è stato istituito presso l'Azienda Ospedaliero – Universitaria di Bologna, Policlinico S.Orsola-Malpighi con delibera n. 6 del 10/1/2018. Il CE-AVEC opera in conformità al DM 12/05/2006 e al DM 08/02/2013.

Dr. Stefano Benini
UOC Governo Clinico e Sistema Qualità

Oggetto: Progetto FATICHE E RISORSE NEI CAREGIVER DI PERSONE CON SCLEROSI MULTIPLA. UNO STUDIO QUALITATIVO – Posizione del CE-AVEC

Gent.mo,

in merito alla sua email del 24 febbraio 2021, si conferma che il progetto in oggetto non necessita un parere da parte del CE-AVEC essendo uno progetto qualitativo che non tratta dati personali.

Cordiali saluti,

Corrado Iacono
Segreteria CE-AVEC per l'
AUSLBO-IM
