# Supplementary material for: Burden and resources in caregivers of people with multiple sclerosis: A qualitative study
Source: PLoS One. 2023 Apr 17;18(4):e0265297. doi: 10.1371/journal.pone.0265297 (PMC10109507; doi:10.1371/journal.pone.0265297)
Supplement: Attachment — Submitted filename: 221229 PONE-D-22-05892 RESPONSE TO REVIEWERS (3).docx [file pone.0265297.s002.docx]

**RESPONSE TO REVIEWERS**

Reviewers' comments:

Reviewer's Responses to Questions

**Comments to the Author**

Reviewer #1: The paper is readable and clear. Thanks for the opportunity of reading about this topic! I want to thank the authors for this exciting work. However, some points should be considered to revise the manuscript.

**Methods:
We invite the authors to justify their decision of methodology in further detail. Numerous other methodologies have been used in caregiver research and thus your justification is not suitable. The implications of this approach are warranted**.

We thank the reviewer for the request. We have better explaind our chice in the methods section

**What does 'All protocol requirements have been formulated with respect for data confidentiality and
34 confidentiality required by the applicable regulatory regulations." mean?**
We thank the reviewer for highlighting the need to clarify this point. We have rewritten the sentence more concisely and clearly. We intended to certify that all regulations on data protection had been thoroughly applied.

**The analysis section needs more detail.**

The study is a qualitative work. We have now added more details about the analytical method, adding a sentence at the end of this section: “…The research team used several strategies to ensure the accuracy of the findings. The analytic team met regularly throughout the iterative process of data analysis to reflect on ways individual values, experiences, or preconceptions might influence or create bias in interpretations of the data may have informed perceptions of participant statements and emerging themes. In addition, the analytic team wrote memos throughout the data analysis, noting observations and reactions to the interview responses. Finally, the last author (AL), who had not previously participated in data analysis, conducted an independent review of the findings and coding documents to assess the trustworthiness of the analysis.”

**Results
Why are these presented as bullet points? A more throughout thematic analysis of the burden of assistance and the effect of the effort is needed.**

Rather than bullet points it was a sequence of the 6 identified relevant themes analyzed from the transcripts of the interviews and focus group. We have maintained, therefore, the division into the main themes emerged from interviews and for each we have tried to extract and cite verbatim meaningful parts of the interviews. We have also tried to deepen the considerations about the meaning of the different issues raised by caregivers as requested.

**Overall:
I think a review for colloquial language should be conducted**

We thank reviewer #1 for highlighting this point. We have revised language accordingly, and we hope this will satisfy reviewers and editor. However colloquial expressions have been maintained in excerpts from interviews.

**Reviewer #2: This is an interesting topic focusing on the burden of caregiving and exploring further what that burden might look like. There is also a brief evaluation of the MS clinical pathway and the local support group.
At the moment, the rationale for the study could be stronger in relation to the gap in the literature and also the positioning of the study in relation to the evaluation context – is this really an evaluation of the MS clinical pathway? If it is then there needs to be a much closer tie between the facets of burden identified and the ways in which the MS clinical pathway does/does not meet those needs currently (maybe a table would help here?)**
We thank the reviewer for raising the issue of the evaluation of the clinical pathway. After thorough consideration we have opted for not evaluating the CPW, only mentioning some issues raised by caregivers. We have amended the manuscript eliminating point b from the last paragraph of the introduction.

**• The rational needs to be clearer. Old studies are cited but what has significantly changed in the past 20 years that suggests that an update is necessary – I agree it might be the context needs to be made explicit.**

We thank the reviewer for this comment. We have updated the literature and hope to have better clarified the rationale.

**• If this is going to include an evaluation of the MS clinical pathway then we need to hear more about it – we need some context about what it is/its aims etc**

As stated above we have decided to limit the study to cargivers observations. A further study, in the future, might be dedicated to a formal evaluation of changes needed to improve the efficacy of the CPW and improve caregivers satisfaction. A yearly monitoring and revision of the CPW is performed in this respect

**• What is meant in this context by ‘resources’ - groups, family and friends, online information etc? This should be described further with relevant literature on what we already know about these resources included.**

We have added a sentence in the methods section: b) assess which resources the carers rely on to manage the burden of care. These include: psychological help, emotional resources; relationship (family, friends or groups); support system resources, financial resources etc.

**Method**
• **Justify excluding early stage caregivers**

We thank the reviewer for raising this point. In MS caregivers are not needed in the early stages of Multiple Sclerosis, where the patient may have subtle impairment of some functions, but usually does not need a caregiver. Thus we did not exclude caregivers of people in the early stages of the disease, who were very few, if any. We have concentrated our attention on PwMS at stages where impairment and disability are more pronounced and require a caregiver
• **What does AUSL stand for?**

We thank the reviewer for asking. This is the acronym for Azienda Unica Sanitaria Locale, which identifies the Local Health Authority of Bologna, responsible for Healthcare provision in the area of Bologna, in the Emilia Romagna Region, in Italy.
**• What is the survey – do you mean the interview schedule?**

We thank the reviewer for suggesting a more precise wording.

**• It would be useful to have more information around the interview schedule and some example questions? Did these vary for the focus groups?**

We thank the reviewer for asking for more information. We have added the required information in the manuscript. In particular the same questions were asked both in interviews and in the single focus group
• **How many focus groups were there?**

We thank the reviewer for asking. We held a single focus group, due to the small number of subjects included in the study and willing to participate to a focus group. People unable or unwilling to attend the focus group were individually interviewed

**• Describe the characteristics of the caregivers (the participants) first before describing the pwMS and refer to table 1 in the text.**

We thank the reviewer for noticing that table 1 was unlinked. We have now amended this imprecision and modified the order of presentation of caregiver and patient characteristics according to the reviewer suggestions.

**• What does EDSS stand for?**

We apologize for the use of a technical acronym without explaining first its meaning

The EDSS is the Expanded Disability Status Score, a scale used to assess impairment (below 4) or disability (4 or higher). The score derives from the scores in 7 different “functional systems”: pyramidal, sensory, brainstem, cerebellar, visual, mental, bowel and bladder. It’s not arithmetical, 0 stands for normal neurological examination, 10 for death due to MS. In between relevant scores for this study: 6 stands for needing a stick, 7 needing a wheelchair (Kurtzke, 1983:

Kurtzke JF. Rating neurologic impairment in multiple sclerosis: an expanded disability status scale (EDSS). Neurology. 1983 Nov;33(11):1444-52. doi: 10.1212/wnl.33.11.1444. PMID: 6685237).

**• More information on the analysis is required -how was this conducted, which authors were involved?**
Authors involved in interviews and the focus group were SB and EP. CD provided critical methodological supervision and AL discussed the results with the other co-authors and revised the draft. Details have now been reported in the last paragraph of the analysis section.

**Results and discussion**
• **The themes mentioned in the opening paragraph of the results section do not match the themes presented beneath. Different number (4 then 6) in a different order and with different names. This needs to be reviewed.**

We thank the reviewer for noticing the discrepancies between the methods section and the results section. We have revised the text accordingly

• **The first theme is interesting and speaks to the complexity of ‘burden’ but it feels a little rushed and under explored here. Can you use longer, richer quotes?**

Thank you for asking longer quotes, we had omitted for brevity. We have now widened the quotations

**• Quotes need to be attributed to P1, P2 etc.**

We have now attributed quotes to individual caregivers, in the table called PARTICIPANTS, therefore in brief P (P01 to P17)

**• Some themes are very descriptive in nature and are list like in places and some contain very few or no quotes to support the interpretation.**

We have increased quotations in order to provide a better support for themes identified

**• Overall, this section feels unbalanced. The first theme feels like a theme with a lot of potential for really exploring the different facets of burden, the other themes feel either very light weight with few supporting quotes or more like a straightforward evaluation of the service. I think the paper needs to emphasise either the themes of burden further or at least find a way to tie together the burden and the current resources/support of the service to bring the findings together and identify key gaps for service provision and for future research. This could be achieved in the results and also carried through into the discussion.**

This is a very relevant observation. We hope the revised version of the results and discussion sections will be found satisfactory

6. PLOS authors have the option to publish the peer review history of their article ([what does this mean?](https://journals.plos.org/plosone/s/editorial-and-peer-review-process#loc-peer-review-history)). If published, this will include your full peer review and any attached files.


If you choose “no”, your identity will remain anonymous but your review may still be made public.


**Do you want your identity to be public for this peer review?** For information about this choice, including consent withdrawal, please see our [Privacy Policy](https://www.plos.org/privacy-policy).

Reviewer #1: No

Reviewer #2: No

[NOTE: If reviewer comments were submitted as an attachment file, they will be attached to this email and accessible via the submission site. Please log into your account, locate the manuscript record, and check for the action link "View Attachments". If this link does not appear, there are no attachment files.]

While revising your submission, please upload your figure files to the Preflight Analysis and Conversion Engine (PACE) digital diagnostic tool, <https://pacev2.apexcovantage.com/>. PACE helps ensure that figures meet PLOS requirements. To use PACE, you must first register as a user. Registration is free. Then, login and navigate to the UPLOAD tab, where you will find detailed instructions on how to use the tool. If you encounter any issues or have any questions when using PACE, please email PLOS at [figures@plos.org](mailto:figures@plos.org). Please note that Supporting Information files do not need this step.
