# Supplementary material for: Burden and resources in caregivers of people with multiple sclerosis: A qualitative study
Source: PLoS One. 2023 Apr 17;18(4):e0265297. doi: 10.1371/journal.pone.0265297 (PMC10109507; doi:10.1371/journal.pone.0265297)
Supplement: Attachment — Submitted filename: PONE-D-22-05892R2 RESPONSE TO REVIEWERS.docx [file pone.0265297.s003.docx]

**RESPONSE TO REVIEWERS**

PONE-D-22-05892R1

BURDEN AND RESOURCES IN CAREGIVERS OF PEOPLE WITH MULTIPLE SCLEROSIS: A QUALITATIVE STUDY

PLOS ONE


Point-by-point response

**1. Clearly provide details of the Ethical Review Board the protocol was submitted to**

We have added in the text what was already attached to the submission: the local ethics committee is the

Comitato Etico di Area Vasta Emilia Centro della Regione Emilia-Romagna (CE-AVEC), and was active since

10/1/2018. As stated in Italian, there is no need to submit to the ethics committee, as no personal details have been provided

**2. Check journal requirements for labelling tables**

We have adhered to journal requirements (Caption above the table). We apologize for not noticing earlier.
**3. Remove bullet points in discussing 'Theme a'**

We have removed the bullet points, numbering in the text the 6 issues
**4. Change the word to 'Limitations' on p13**

We have changed limits to LIMITATIONS, as required
**5. Define the appropriate referencing style in line with journal requirements and ensure consistency. Each reference is presented differently from the next.**

We apologize for the imprecisions. References have been revised to be in line with journal requirements

In addition we have corrected some typos we picked in re-reading the manuscript.

==============================

**Journal Requirements:

Please review your reference list to ensure that it is complete and correct. If you have cited papers that have been retracted, please include the rationale for doing so in the manuscript text, or remove these references and replace them with relevant current references. Any changes to the reference list should be mentioned in the rebuttal letter that accompanies your revised manuscript. If you need to cite a retracted article, indicate the article’s retracted status in the References list and also include a citation and full reference for the retraction notice.**
We have not cited papers which have been retracted. A few are not on PubMed, but have a DOI, which we have used to enable readers to easily find the right article or chapter in a book (they are open access).

**Additional Editor Comments (if provided):**

**1. Clearly provide details of the Ethical Review Board the protocol was submitted to
2. Check journal requirements for labelling tables
3. Remove bullet points in discussing 'Theme a'
4. Change the word to 'Limitations' on p13
5. Define the appropriate referencing style in line with journal requirements and ensure consistency. Each reference is presented differently from the next.**

See above

[Note: HTML markup is below. Please do not edit.]

Reviewers' comments:

Reviewer's Responses to Questions

**Comments to the Author**

1. If the authors have adequately addressed your comments raised in a previous round of review and you feel that this manuscript is now acceptable for publication, you may indicate that here to bypass the “Comments to the Author” section, enter your conflict of interest statement in the “Confidential to Editor” section, and submit your "Accept" recommendation.

Reviewer #2: All comments have been addressed

Reviewer #3: (No Response)

2. Is the manuscript technically sound, and do the data support the conclusions?

The manuscript must describe a technically sound piece of scientific research with data that supports the conclusions. Experiments must have been conducted rigorously, with appropriate controls, replication, and sample sizes. The conclusions must be drawn appropriately based on the data presented.

Reviewer #2: Yes

Reviewer #3: Yes

3. Has the statistical analysis been performed appropriately and rigorously?

Reviewer #2: N/A

Reviewer #3: Yes

4. Have the authors made all data underlying the findings in their manuscript fully available?

The [PLOS Data policy](http://www.plosone.org/static/policies.action#sharing) requires authors to make all data underlying the findings described in their manuscript fully available without restriction, with rare exception (please refer to the Data Availability Statement in the manuscript PDF file). The data should be provided as part of the manuscript or its supporting information, or deposited to a public repository. For example, in addition to summary statistics, the data points behind means, medians and variance measures should be available. If there are restrictions on publicly sharing data—e.g. participant privacy or use of data from a third party—those must be specified.

Reviewer #2: Yes

Reviewer #3: Yes

5. Is the manuscript presented in an intelligible fashion and written in standard English?

PLOS ONE does not copyedit accepted manuscripts, so the language in submitted articles must be clear, correct, and unambiguous. Any typographical or grammatical errors should be corrected at revision, so please note any specific errors here.

Reviewer #2: Yes

Reviewer #3: Yes

6. Review Comments to the Author

Please use the space provided to explain your answers to the questions above. You may also include additional comments for the author, including concerns about dual publication, research ethics, or publication ethics. (Please upload your review as an attachment if it exceeds 20,000 characters)

Reviewer #2: (No Response)

Reviewer #3: 1. Ethical considerations: Clearly provide details of where the protocol was submitted for review (and found that no authorization was necessary)
2. Check journal rules concerning labelling tables
3. Results: Present findings of 'Theme a' without bullet points.
4. Change the word to 'Limitations' on p13
5. References: Define the referencing style and ensure consistency. Each reference is presented differently from the next.

7. PLOS authors have the option to publish the peer review history of their article ([what does this mean?](https://journals.plos.org/plosone/s/editorial-and-peer-review-process#loc-peer-review-history)). If published, this will include your full peer review and any attached files.


If you choose “no”, your identity will remain anonymous but your review may still be made public.


**Do you want your identity to be public for this peer review?** For information about this choice, including consent withdrawal, please see our [Privacy Policy](https://www.plos.org/privacy-policy).

Reviewer #2: No

Reviewer #3: No

[NOTE: If reviewer comments were submitted as an attachment file, they will be attached to this email and accessible via the submission site. Please log into your account, locate the manuscript record, and check for the action link "View Attachments". If this link does not appear, there are no attachment files.]

While revising your submission, please upload your figure files to the Preflight Analysis and Conversion Engine (PACE) digital diagnostic tool, <https://pacev2.apexcovantage.com/>. PACE helps ensure that figures meet PLOS requirements. To use PACE, you must first register as a user. Registration is free. Then, login and navigate to the UPLOAD tab, where you will find detailed instructions on how to use the tool. If you encounter any issues or have any questions when using PACE, please email PLOS at [figures@plos.org](mailto:figures@plos.org). Please note that Supporting Information files do not need this step.

*In compliance with data protection regulations, you may request that we remove your personal registration details at any time.*  [*(Remove my information/details)*](https://www.editorialmanager.com/pone/login.asp?a=r)*. Please contact the publication office if you have any questions.*
